# Reductions in Artemisinin-Based Combination Therapy Consumption after the Nationwide Scale up of Routine Malaria Rapid Diagnostic Testing in Zambia

**DOI:** 10.4269/ajtmh.2012.12-0127

**Published:** 2012-09-05

**Authors:** Joshua O. Yukich, Adam Bennett, Audrey Albertini, Sandra Incardona, Hawela Moonga, Zunda Chisha, Busiku Hamainza, John M. Miller, Joseph Keating, Thomas P. Eisele, David Bell

**Affiliations:** Tulane University School of Public Health and Tropical Medicine, New Orleans, Louisiana; Foundation for Innovative New Diagnostics (FIND), Geneva, Switzerland; Ministry of Health, National Malaria Control Centre, Lusaka, Zambia; Akros Research, Lusaka, Zambia; PATH Malaria Control and Evaluation Partnership in Africa (MACEPA), National Malaria Control Centre, Lusaka, Zambia

## Abstract

The National Malaria Control Center of Zambia introduced rapid diagnostic tests (RDTs) to detect *Plasmodium falciparum* as a pilot in some districts in 2005 and 2006; scale up at a national level was achieved in 2009. Data on RDT use, drug consumption, and diagnostic results were collected in three Zambian health districts to determine the impact RDTs had on malaria case management over the period 2004–2009. Reductions were seen in malaria diagnosis and antimalarial drug prescription (66.1 treatments per facility-month (95% confidence interval [CI] = 44.7–87.4) versus 26.6 treatments per facility-month (95% CI = 11.8–41.4)) pre- and post-RDT introduction. Results varied between districts, with significant reductions in low transmission areas but none in high areas. Rapid diagnostic tests may contribute to rationalization of treatment of febrile illness and reduce antimalarial drug consumption in Africa; however, their impact may be greater in lower transmission areas. National scale data will be necessary to confirm these findings.

## Introduction

Malaria continues to be a key focus of public health services in Zambia, with a large scale up of antimalarial interventions and considerable progress in control of the disease in recent years.^1^,^2^ In 2009, 2.9 million cases and nearly 3,862 malaria-attributable deaths were reported in a population of 12 million, down from 3.3 million reported cases and 9,369 deaths.^1^ In addition to insecticide-treated mosquito nets (ITNs) and indoor residual spraying (IRS) in selected areas, resources for malaria control in Zambia have been directed toward more effective, artemisinin-based combination therapies (ACTs) such as artemether-lumefantrine (Coartem). In 2004, Zambia began to progressively introduce ACT in response to growing and widespread resistance to sulfadoxine-pyrimethamine (SP) and chloroquine (CQ), with full national scale reached by early 2005. Sulfadoxine-pyrimethamine continues to be stocked for intermittent preventive therapy in pregnancy (IPTp). At the time of the shift to ACT much diagnosis was still based on non-specific symptoms rather than confirmed malaria parasitemia; consequently, actual malaria incidence at health facilities remained unquantified and many antimalarial treatments were likely misdirected. Faced with poor malaria surveillance data caused by inadequate laboratory diagnosis in most parts of the country, coupled with substantial higher cost of Coartem compared with CQ and SP, the National Malaria Control Center (NMCC) introduced rapid diagnostic tests (RDT) (*ICT Malaria Pf* (ICT Diagnostics, Capetown, South Africa) and *Paracheck Pf* (Orchid Biomedical Systems, Verna, Goa, India) to detect *Plasmodium falciparum* as a pilot in some districts in 2005 and 2006; scale up at a national level was achieved in 2009, when Zambia made the transition to a strategy of full reporting and treatment using only parasitologically confirmed diagnoses nationwide. Laboratory confirmation is now mandated, where capacity exists, before antimalarial treatment, though in some cases stockouts of reagents or test kits may prevent full enactment of the policy.^3^

Microscopy is considered the primary form of diagnosis, whereas RDTs are meant to be used in all situations where microscopy is not available. Since 2009, RDT supplies have been sufficient to stock most health facilities in the country, even those with pre-existing laboratory services, to aid in diagnosis and to meet demand for testing services. Furthermore, RDTs and ACT have also been used outside the health facility in communities since 2007, where community health workers have been trained in malaria diagnosis and treatment. However, stockouts do continue to occur and treatment may continue to be based on clinical diagnosis in some situations.

The use of RDTs at clinic and community levels has the potential to provide accurate malaria diagnosis, restricting antimalarial therapy to those who require it and avoiding the overuse of antimalarial drugs, while enabling more appropriate management of non-malarial fever.^4^ However, these gains will only occur if the RDTs are used consistently and correctly, and if the test results are used by clinicians to inform case management. Some published studies indicate that overall consumption of ACT and other antimalarials decrease after RDT introduction^5^–^7^; conversely, a number of published studies have suggested that expansion of laboratory confirmation (either by microscopy or RDT) may not significantly impact diagnosis and antimalarial drug prescribing practices in sub-Saharan Africa, including in Zambia. This lack of impact on diagnosis and prescription practices may be caused by poor quality of diagnostics, stock outs of essential items, or clinicians' failure to trust the results of tests.^8^–^13^

The use of test results after a large-scale introduction of RDTs may differ from those seen in smaller scale trials, as training and supervisory characteristics likely differ. Additionally, clinicians may respond to results differently if their peers are also routinely using parasite-based diagnosis or after an extended period of exposure to the idea of demonstration of parasites before treatment. In view of the continuing scale-up of RDT use in Zambia and contradictory indications of adherence by providers, we collected data on RDT use, drug consumption, and diagnostic results in three Zambian health districts to determine the impact RDTs were having on malaria case management over the period 2004–2009. Here, we provide a descriptive analysis of these data.

## Materials And Methods

### Study areas and facility selection.

**Figure 1.**
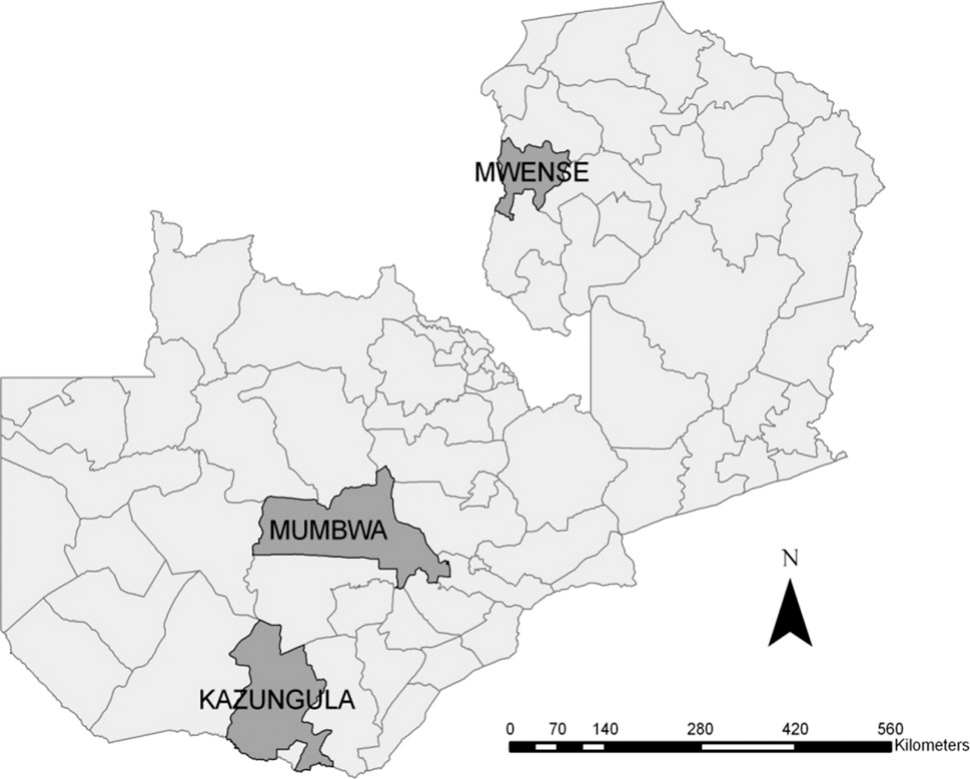
Location of study districts in Zambia.

Three districts: Kazungula, Mumbwa, and Mwense, in southern, central, and northern Zambia, respectively (Figure 1), were selected for this study. The districts cover a range of transmission intensity. Kazungula shares a southern border with Zimbabwe, adjacent to the Zambezi River, and has relatively low transmission; Mumbwa lies almost directly to the north and is also a low transmission area. Mwense District, located in northern Zambia, shares a border with the Democratic Republic of the Congo, and is considered a high malaria transmission area. These districts were among the first to receive RDTs, and record keeping in health facilities was considered by the NMCC to be more complete than observed elsewhere. Similar to other health systems, a hierarchy of health facilities exists ranging from district hospitals, level 1 hospitals (with medical and nursing staff), health clinics (including trained nursing staff), and health posts and community health workers.

In each district, all health facilities that exclusively used RDTs for parasitologically confirmed diagnosis were included, based on information received from district staff. The study was restricted to health clinics, where most suspected malaria cases present. Data from 25 health facilities in total were included in the sample. Data were initially collected from 43 health facilities from January 2004 onward (12 rural health clinics in Kazungula, 2 urban and 20 rural health clinics in Mumbwa, and 9 rural clinics in Mwense). Record-keeping in some facilities was poor, meaning that some facilities had records that were too sparse to include in the analysis. We excluded health facilities from the study if at least one of the four following indicators had a lack of data for four or more consecutive months, throughout the entire study period: “total number of malaria cases,” “number of persons tested by RDT,” “number of persons positive by RDT,” “number of ACT, SP, and other antimalarial treatments.” A number of facilities had records that became sufficiently complete only after 2004; these facilities were included in the analysis from the start of their first 4 months of consecutively available data. After review, only 25 of these proved sufficiently complete to be accepted for analysis and inclusion in this study.

### Data collection.

Because of inadequacies in the nationally aggregated health management information system (HMIS), data collection and reporting in much of Zambia over our study period were based on a hard copy of clinic data obtained directly from each of the facilities, rather than from centralized collated data. All data were collected by transcribing information directly from clinic log books and registers into Microsoft Excel (Microsoft Corp., Redmond, WA). The following indicators were targeted:

Total number of consultations as recorded in *HMIS disease aggregation* at the facility level or the out-patient department (*OPD*) *register*;
Total number of malaria cases (confirmed and suspected) as recorded in *HMIS disease aggregation* at the facility level or the *OPD register*;
Number of persons tested by RDT as recorded in the *Laboratory* or *RDT register*;
Number of persons positive by RDT as recorded in the *Laboratory* or *RDT register*, or Notifiable Disease register;
Number of ACT, SP, and other antimalarial treatments *prescribed*, as *recorded* in the OPD register;
Total number of OPD consultations, as recorded in the *HMIS disease aggregation* at the facility level or the *OPD register*.

All data were collected on a monthly basis from January 2004 to August 2009. The OPD register line listings were used to validate aggregate disease totals for 1 month per year from 2004–2009 for the previous indicators. Sub-samples of clinic registers were digitally photographed for validation at a central level with data transcribed during field work. Further validation consisted of recalculating each indicator using individual patient records recorded in facility registers.

Age aggregations were based on the previous standard of < 5 years of age and ≥ 5 years of age for all indicators collected, with the exception of SP. The SP treatments were disaggregated based on the age group receiving treatments, including newborns < 2 months of age and 2 months of age and above. Treatments to pregnant women were excluded because it was not possible to distinguish SP given as part of the IPTp program from treatment of suspected malaria. Treatments of SP to anyone over 2 months of age were excluded from the malaria treatment category after the roll-out of ACTs to each individual facility, as SP was no longer considered an appropriate treatment.

### Data analysis.

Data were summarized by facility and normalized by the number of outpatients attending each facility; this was done to control for fluctuations resulting from varying facility usage trends. Average rates across districts and the entire sample are thus presented, weighting each facility equally; additional analysis using OPD attendance weighted averages across facilities gave similar results (data not shown). Ratios of confirmed cases to treated cases and tested cases to treated cases are presented in a similar manner; weighting by patient load led to similar results. Malaria diagnoses were defined as all patients given a malaria diagnoses by a clinician, regardless of parasitological confirmation. Proportional malaria morbidity is defined as the fraction of all patients presenting at a facility in a given facility-month who were given a malaria diagnosis. Confirmed cases were defined as patients with a positive RDT test; test positivity rates (TPR) were calculated as the number of patients in a given facility with a positive RDT result during a given month divided by the number of patients who received a RDT test during the same month at the same facility. Ratios of confirmed cases to ACT treatments were calculated by dividing the number of ACT treatments delivered in a facility-month by the number of confirmed cases (RDT positive) during the same facility-month in each facility. This ratio should be equal to one if the treatments are only delivered to each confirmed case and all suspected malaria cases are tested with RDTs. Ratios of tested patients to ACT treatments delivered were calculated by dividing the number of ACT treatments delivered in a given facility-month by the number of patients tested during the same facility-month for each facility. This ratio should be < 1 if all suspected cases are tested and the providers deliver treatments only to RDT-positive patients. Without laboratory confirmation one would expect to see a number of treatments roughly equivalent to the number of suspected (or clinically diagnosed) cases of malaria, although after RDT rollout with perfect implementation one would expect the number of treatments to be equal to the number of suspect cases multiplied by TPR. Significance testing was conducted using Wald tests after adjusting for clustering at the facility level. All statistical analyses were conducted in STATA 11.2 (StataCorp, College Station, TX).

## Results

Reductions were seen overall in the levels of malaria diagnosis and antimalarial drug prescribed pre- and post-RDT introduction. These differences vary between districts, with the two lower malaria transmission districts, Kazungula and Mumbwa, showing large reductions in the frequency of malaria diagnosis and treatment, whereas Mwense district showed a marginally significant increase in treatment post-RDT rollout (95.7 versus 129.0; *F*(1, 24) = 4.09, *P* = 0.0544) (Table 1). After RDT introduction, clinical malaria diagnosis remained higher than parasitologically-confirmed diagnoses in all three districts; however, the number of treatments delivered was lower than the number of malaria diagnoses and more in line with the number of confirmed cases than with the overall number of clinical malaria diagnoses. Rates of malaria diagnosis and ACT treatments per facility-month fell significantly in two of the three districts after the introduction of RDTs (diagnoses: Kazungula 134.0 versus 29.2, *F*(1, 24) = 10.38, *P* = 0.0038; Mumbwa: 213.6 versus 36.2, *F*(1,24) = 8.28, *P* = 0.0083; Treatments: Kazungula: 65.2 versus 12.2, *F*(1, 24) = 4.69, *P* = 0.0405; Mumbwa: 54.7 versus 11.1, *F*(1, 24) = 84.42, *P* < 0.001).

**Figure 2.**
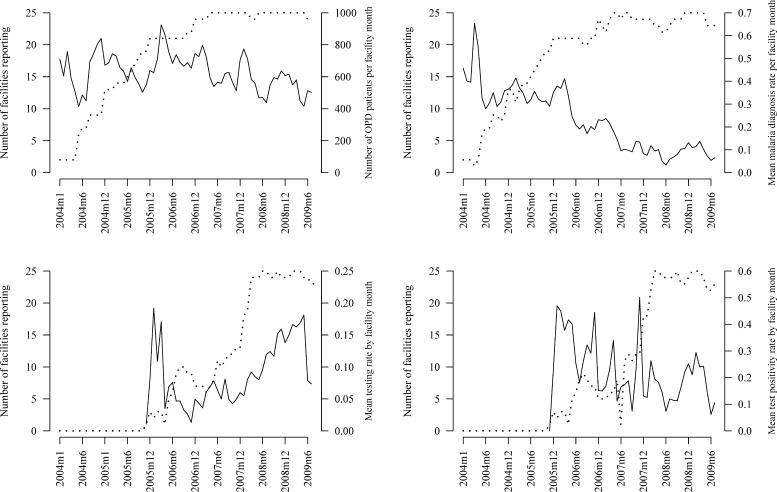
Trends in outpatient visits, malaria diagnosis, testing rate, test positivity rate and reporting completeness at study clinics. (Average value of the indicator across reporting facilities is shown as a solid line; the number of facilities reporting is shown with dotted line referring to left vertical axis in all figures.)

As the number of facilities with sufficiently complete records increased, the reporting rate of laboratory-confirmed malaria in facilities using RDTs fell rapidly, suggesting that the early high mean TPR may be biased by preferential reporting from facilities with higher TPR overall, or by earlier roll-out of RDTs in places that were *a priori* perceived as being high malaria TPR areas. Testing rates were high (close to 20% of total OPD attendance) in these facilities after introduction of RDTs, and dropping to 5% overall before slowly rising again after the number of facilities reporting increased (Figure 2). Once more than 15 facilities were reporting a steady decline in reported malaria was observed, though the testing rate rose and remained high. Total OPD average attendance also slowly declined once the number of facilities reporting increased.

**Figure 3.**
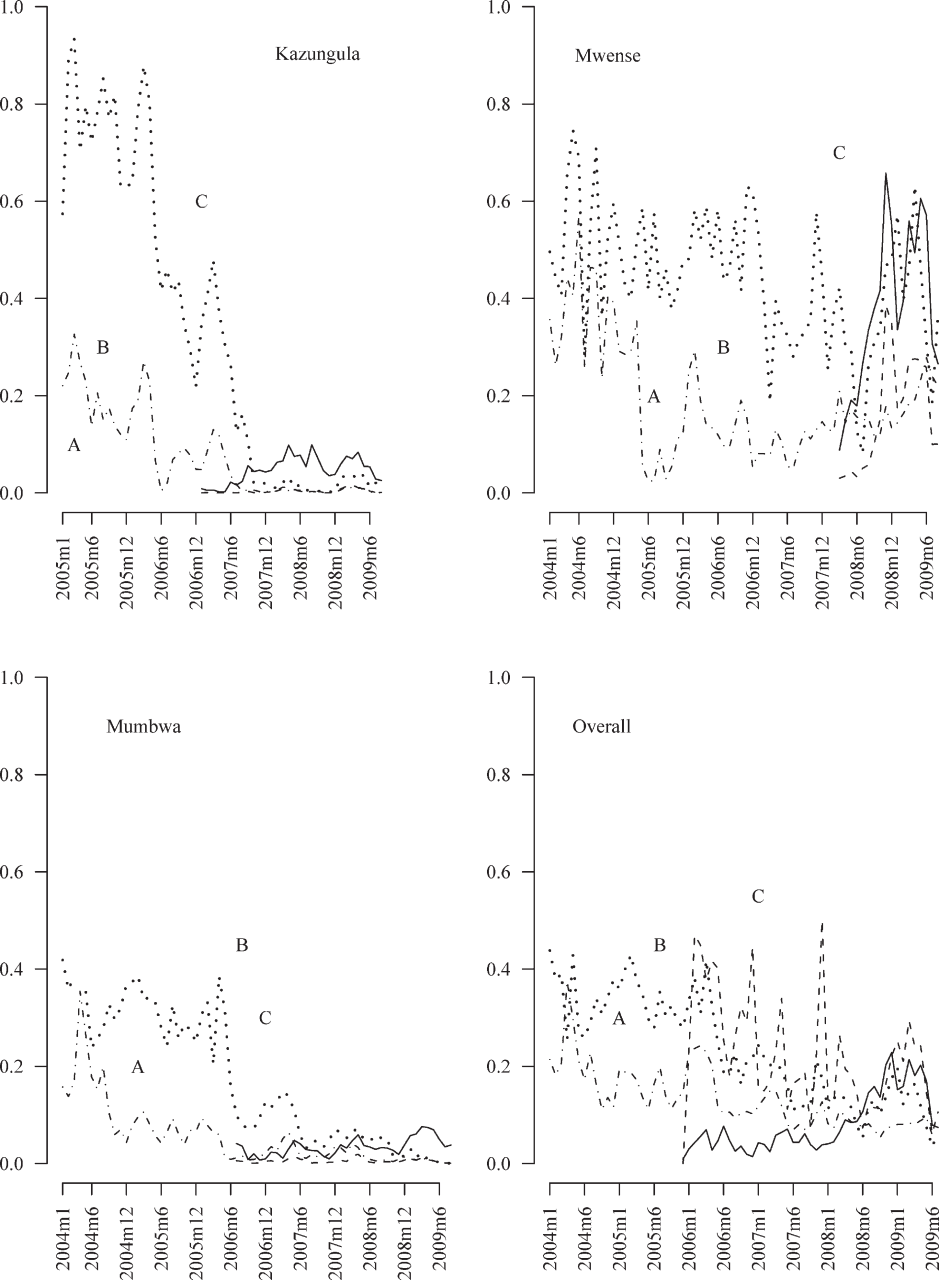
Rates of testing, prescription, and malaria morbidity by district and overall. (Tested shown with solid line, confirmed cases with a dashed line (- - -), treated patients with a dotted and dashed line (- · -), and all malaria diagnoses shown with a dotted line ( ⋯ ). The **A** indicates the start of free insecticide treated net availability, the **B** indicates the average start of ACT use and the **C** indicates the average start of RDT use.)

The fraction of all OPD patients receiving nationally recommended antimalarial drugs fell markedly over the 6-year time period in Kazungula and Mumbwa, remaining low since late 2007 (Figure 3). Similar trends are seen in proportional malaria morbidity among all patients and TPR among those who were tested by RDTs. The testing rate has gradually increased over time with a corresponding reduction in total recorded malaria morbidity (incidence of cases identified as malaria by the health facility both with and without laboratory confirmation) and consumption of antimalarial drugs. As expected, although laboratory testing rates have gradually increased over time and remain relatively stable, TPRs show a highly seasonal pattern with increased positivity during the malaria transmission season.

**Figure 4.**
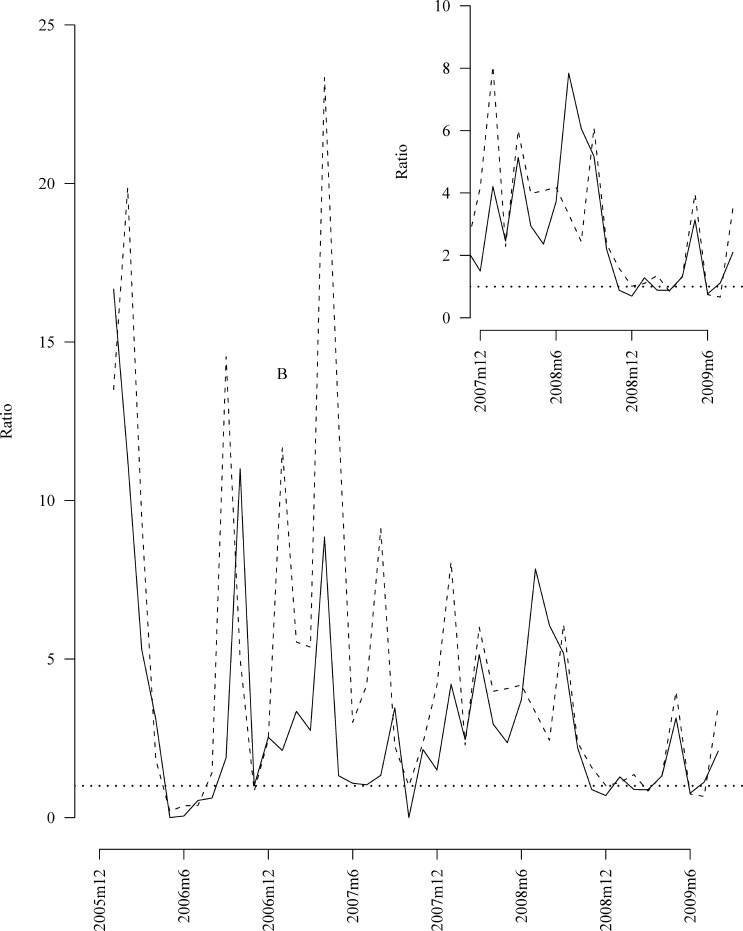
Ratio of treatment with ACT to confirmed malaria cases over time. (The solid line represents patients under five years of age the dashed line patients over five years of age and the dotted horizontal line the ideal ratio. The **B** indicates the average start-time of RDT use.)

Figure 4 shows the ratio of treatments to confirmed malaria cases over time. Although this ratio overall remained much higher than one after RDTs, by the start of 2009 it had stabilized around one, meaning that treatments were not being administered in excess of the numbers of confirmed cases seen at facilities. (Note: If only patients with laboratory confirmed malaria cases were treated with antimalarials this ratio would be expected to be one.)

The ratio of treatments to tested individuals fell over time (data not shown). Ideally, all potential malaria patients should be tested preceding treatment, meaning that the ratio of ACT prescriptions to tests should fall below one and should mirror the TPR. These rates appear to have varied early in the process of RDT introduction, but stabilized over time below one and are in line with TPR values.

**Figure 5.**
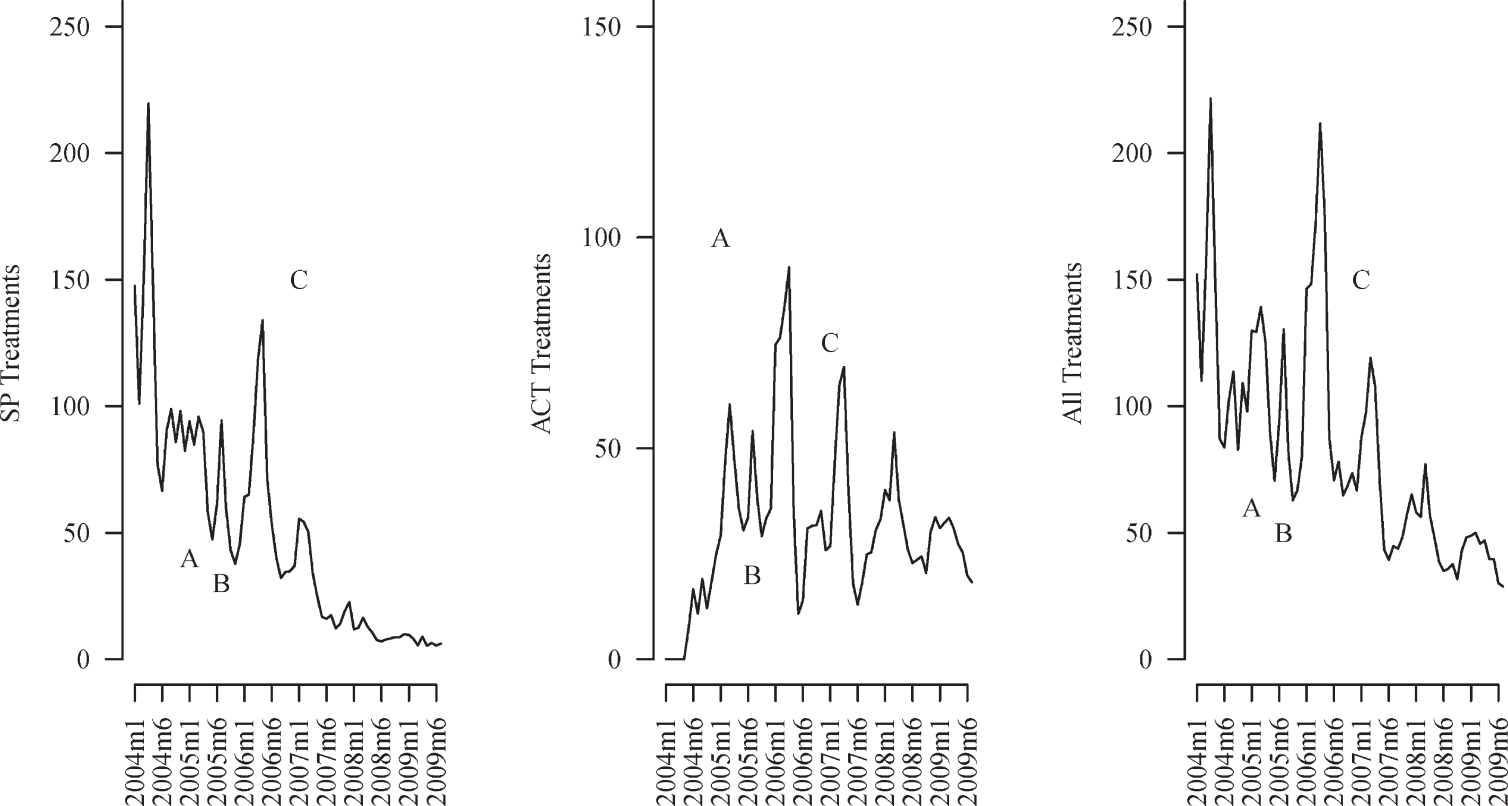
Mean treatments over time at study facilities. (The **A** indicates the start of free insecticide treated net availability, the **B** indicates the average start of ACT use and the **C** indicates the average start of RDT use.)

Trends in absolute malaria diagnosis at facilities and in absolute prescription practices reflected the trends seen in data normalized for OPD attendance (data not shown). Figure 5 shows trends in SP, ACT, and all antimalaria treatments over time regardless of whether they met national guidelines, but excluding doses of SP given for IPTp. Generally, these figures show a decreasing trend in the delivery of antimalarial drugs over time. The ACT prescriptions initially increase during the national switch to ACTs, but then begin to fall as diagnosed malaria cases decrease with RDT rollout. We cannot be sure that such trends are not attributable to secular trends arising from potential confounding factors. Before the switch to RDTs the average facility delivered ∼66 antimalarial treatments per month; post roll-out of RDT prescription rates fell significantly, to below 27/month (adjusted Wald statistic, *F*(1,24) = 16.60, *P* < 0.001). Figure 5A reflects not only general decreasing treatment delivered over time but also a national policy switch in first-line treatment policy from SP to ACTs. These gains are driven by changes in the two districts of Mumbwa and Kazungula, whereas Mwense district showed a change in ACT treatments in the opposite direction.

Proportional malaria morbidity in Mwense district was high both pre- and post-RDT rollout with 17% of patients receiving a malaria diagnosis before RDTs and 22% after RDTs. During post RDT rollout malaria diagnosis and TPRs remained high. Fifteen percent of patients were diagnosed with laboratory-confirmed malaria post-RDT rollout in this district, and the average TPR across facilities varied between 20% and 60%, whereas TPR in the other two districts remained below 10%.

Data from Mumbwa and Kazungula districts appear to show that reductions in antimalarial use were delayed for some time after RDT introduction. Major reductions in ACT consumption in Mumbwa and Kazungula districts appeared to follow introduction of RDTs by a period of 6 months to one and a half years.

## Discussion

Ideally, when drugs and diagnostics are available, clinicians should request tests for each febrile patient and follow the results of these laboratory tests with standard of care prescribing practices. Thus, the ratio of ACT doses to confirmed malaria cases should be close to one. Figure 4 illustrates that this ratio, although varying highly over time, has gradually stabilized near one after the introduction of RDTs. Although deviations from best practices clearly still continue, their magnitude appears to be much smaller as RDTs have been present in facilities for longer periods.

Laboratory confirmation of parasite infection is important to management of malaria and other febrile illness because to properly manage febrile illness, especially in the context of changing malaria burden and in lower malaria transmission areas, it is necessary for clinicians to accurately know the actual infection status of each patient. In the past, Integrated Management of Childhood Illness (IMCI) guidelines recommended treatment of all febrile children with an antibiotic and an antimalarial in *Plasmodium falciparum*-endemic settings; currently, the World Health Organization (WHO) recommends confirmatory diagnosis before treatment in all settings where a diagnostic test can be made available. RDTs provide perhaps the only potential diagnostic solution for remote rural health facilities or communities in Africa, and as such, it is important that they are used and trusted by clinicians. Moreover, malaria control programs need accurate information on patient load and infection rates for monitoring and evaluation, as well as for planning prevention and control strategies.

Reductions in consumption of ACTs after the scale up of RDTs clearly occurred in two of the three districts in this study, taking some time to stabilize close to the RDT positivity rate after introduction of RDTs. The third district, Mwense, did not appear to reduce ACT consumption and may in fact have increased consumption during the period for which records were reviewed after RDT rollout. Though ACT treatment and RDT positivity rates were in general agreement, in Mwense District results appeared chaotic. This was probably influenced by stock-outs and incomplete reporting soon after the introduction of RDTs in this district (Figure 3C).

The high levels of malaria in Mwense District may be one reason that reductions in ACT or other antimalarial use was not observed there. Furthermore, the time since RDT introduction in this area was shorter than in the other two districts in this study and largely covered the high transmission period. This further occurred during a year in which the north of Zambia reportedly suffered from increased malaria incidence^14^; given this, gains from RDT introduction in Mwense might be expected to remain smaller than in lower transmission areas. However, there is a possibility that meaningful gains may eventually be realized, given a longer time frame for observation.

Similar improvement in diagnostic and treatment practices, like those seen in Mumbwa and Kazungula districts, coupled with reductions in ACT consumption since the introduction of RDTs have been seen in other African settings. A recent analysis by Thiam and others^6^ of HMIS data in Senegal indicated that after a period of one and a half years after RDT rollout, ACT consumption began to mirror confirmed malaria cases at the national level. In Senegal the treatment of febrile illness with ACTs fell by more than 50%, similar to the results seen here. Controlled trials in mainland Tanzania and Zanzibar^5^,^15^–^18^ also showed dramatic reductions in ACT usage after the introduction of RDTs. Though other trials and modeling studies have indicated that provider non-compliance with test results can lead to significant reductions in the ability of RDT introduction to lead to reductions in ACT consumption and improve health outcomes,^8^,^19^–^21^ this does not appear to be a major problem in the districts of Zambia studied here. One potential concern with the introduction of diagnostics is that patients with false-negative test results, especially young children, will not receive antimalarial drugs and thus be at high risk of proceeding to severe malaria or death.^22^ Although our dataset does not allow us to address this risk, data from other sites suggests that this risk is probably minimal. Most patients with false-negative RDT results likely have low parasitemia levels and as such are unlikely to have severe complications relative to patients with higher parasite loads.^5^,^23^,^24^

Although we cannot directly address provider compliance with test results at an individual patient level using the dataset at hand, we can see that both in high and low prevalence areas post RDT rollout the rates of prescription of ACTs became similar to the rates of parasitologically confirmed cases. This provides possible confirmation that the introduction of RDTs has helped not only to reduce consumption and overuse of ACTs, but also to rationalize treatment practices for febrile illness. More detailed individual level data would be required to verify with certainty that febrile parasite negative patients did not receive antimalarials.

Testing rates in the two low prevalence districts were also low, and it is not clear exactly what clinical criteria were used to select suspected malaria cases for testing, because we do not have data on the number of patients presenting with febrile illness. In Mwense District, where proportionate malaria morbidity has remained high, a much larger fraction of all patients have received laboratory tests. This suggests that providers in Zambia are able and willing to test significant numbers of patients with RDTs even in areas where clinical diagnosis may be a marginally more effective predictor of malaria infection than in low prevalence areas.

Attribution of these results directly to the roll-out of RDTs is not possible because many potential confounding factors exist that cannot be controlled in a descriptive analysis with a comparison to historical data. Moreover, this study did not attempt to establish a counter-factual group, a precursor to definitively establishing impact. The logical reason that RDTs may contribute to changing malaria reporting practices and usage of ACTs is the hope that over time RDTs can change clinical practice and move the case definition of “malaria” (meaning all suspected malaria cases or all fever cases) in routine health systems to a definition of malaria that is routinely based on laboratory-confirmed parasitemia combined with clinical symptoms. Potential confounding factors preventing the attribution of the changes seen over time in these facilities to RDTs include changes in the true malaria burden caused by scale up of ACTs and vector control, changes in the burden caused by other factors including climate or changes in treatment seeking behavior in the catchment areas of the facilities, and changes in reporting practices over time. More investigation including controlling for climatic factors, vector control scale up, and drug treatment practices will be necessary to validate the descriptive results shown here.

Vector control scale-up in Zambia, including the expansion of insecticide-treated bed net coverage and IRS, has been substantial over the past several years. In the framework of an ecological analysis it is not possible to eliminate changes in malaria morbidity resulting from changes in underlying malaria incidence and prevalence trends caused by vector control or weather, from changes because of diagnostics. The large increases in testing and the trend toward concordance between confirmatory tests and drug prescriptions tends to lend credence to the assertion that diagnostics are indeed contributing to the reduced reported incidence of malaria in Zambia over this time period as well as to reductions in the use of drug therapy for malaria. However, more detailed information on the changes in vector control and climate over the time period could help to control for potential confounding and should be incorporated into future research on this topic.

Additionally, incomplete record keeping in facilities limited the possibility to analyze data from all facilities in the three selected districts. If poor usage of RDTs is linked to poor quality reporting our results may overestimate the influence of RDTs on the reduction in ACTs. However, the retrospective nature of the study excludes investigator influence on patterns of adherence, and the results are thus likely to reflect common patterns across the country as a whole. One other potential source of selection bias arises from the exclusion of higher level facilities where microscopy was also in place and staff was likely to be more highly trained. Significant evidence suggests that there may be a negative correlation between level of clinical training and provider adherence to test results for malaria.^25^,^26^ Although this potential bias might lead to an overestimation of the benefits of RDT introduction, the majority of out-patient malaria cases in Zambia present at lower level facilities such as those included in this study. Ultimately the effect of such a selection bias on our results is probably limited. Across the three districts, reported numbers of treatments generally fell below the number of malaria diagnoses, especially during the early periods of data collection. This is likely a result of several factors including stock-outs of drugs and underreporting of treatments as compared with diagnoses. Less pressure for accurate reporting of drug usage was probably present pre-ACT introduction. Although both of these causes of measurement error may introduce bias into the results of the study, the likely direction is toward the null hypothesis that RDTs had no effect on the use of antimalarial drugs. The roll-out of RDTs in these districts of Zambia has coincided with a significant reduction in ACT consumption in low transmission districts. In areas of higher transmission, results were less clear, consumption does not appear to have been reduced however treatment practices may still have benefited from the availability of laboratory diagnostic methods. RDTs may contribute to the rationalization of treatment of febrile illness and reduce ACT consumption in Africa, but are likely to be both more effective and efficient in lower transmission areas. More national scale data will be necessary to confirm these findings.

## Figures and Tables

**Table 1:** Malaria diagnoses and treatment summary statistics

	Kazungula District		Mumbwa District		Mwense District		Total	
	Pre	Post	Pre	Post	Pre	Post	Pre	Post
Total facilities with any report	10	10	10	10	5	5	25	25
Facility months of data	196	312	267	310	124	90	587	712
Total OPD patients per facility month	513.7 (144.8) [215–813]	385.5 (70.2) [241–530]	768.6 (220.6) [313–1224]	722.3 (193.0) [324–1121]	740.1 (221.9) [282–1198]	761.8 (178.9) [393–1131]	678.3* (120.2) [430–926]	580.1* (100.2) [373–787]
RDT tests per facility month	NA	22.8 (3.7) [15.1–30.4]	NA	29.3 (4.3) [20.4–38.1]	NA	289.9 (99.3) [84.9–494.8]	NA	60.6 (20.0) [19.3–101.9]
RDT positives per facility month	NA	2.6 (0.59) [1.4–3.8]	NA	4.8 (1.7) [1.2–8.4]	NA	125.7 (40.0) [43.2–208.3]	NA	19.9 (8.9) [1.6–38.3]
Malaria diagnoses per facility month	134.0† (32.1) [67.6–200.3]	29.2† (11.3) [6.0–52.5]	213.6† (79.6) [49.3–377.9]	36.2† (19.0) [3.1–75.5]	331.1 (110.1) [104.0–558.3]	300.1 (74.2) [146.9–453.3]	212.0† (44.8) [119.5–304.5]	65.3† (18.9) [26.2–104.4]
SP doses per month	50.1† (11.4) [26.5–73.7]	8.0† (3.3) [1.1–14.8]	77.4† (14.4) [47.7–107.1]	12.8† (4.3) [3.9–21.6]	63.2† (17.0) [28.0–98.4]	34.7† (16.9) [×0.1–69.5]	65.2† (8.7) [47.3–83.2	13.5† (3.4) [6.4–20.5]
ACT doses per month	55.3* (22.6) [8.6–102.0]	12.2* (3.8) [4.3–20.0]	28.2† (6.1) [15.7-40.7]	11.1† (3.2) [4.5–17.8]	82.3† (30.5) [19.3–145.3]	129.0† (13.3) [101.5–156.5]	50.2† (12.1) [11.8–41.4]	26.6† (7.2) [11.8–41.4]
Total antimalarials per month	82.8† (16.7) [48.4–117.3]	21.0† (8.2) [4.2–37.9]	104.5† (15.0) [73.5–135.4]	25.5† (8.2) [8.7–42.4]	149.0† (30.0) [87.1–210.9]	185.5† (27.9) [128.0–243.0]	109.3† (11.6) [85.2–133.3]	45.4† (11.3) [22.1–68.8]
ACT/SP doses per facility month‡	62.5†* (23.4) [14.2-110.8]	12.2† (3.8) [4.3–20.0]	54.7† (6.3) [41.7–67.8]	11.1† (3.2) [4.5–17.8]	95.7* (27.1) [39.7–151.6]	129.0* (13.3) [101.5–156.5]	66.1† (10.3) [44.7–87.4]	226.6† (7.2) [11.8–41.4]

* Indicates significant difference at 10% level between pre and post rapid diagnostic test (RDT) period after adjusting for clustering.

† Indicates significant difference at 5% level between pre and post RDT period after adjusting for clustering.

‡ Artemisinin-based combination therapy/sulfadoxine-pyrimethamine (ACT/SP) doses were calculated based on SP treatment doses before a facility-specific switch to ACTs and ACT doses provided after the switch.

Value (Standard Error) [95% Confidence Interval]. OPD = out-patient department.
